# Professional Observations for the Year 1837

**Published:** 1836

**Authors:** 


### II.—Professional Observations for the year 1837.

The month which is closing the year 1836, has been remarkable, for its great and repeated vicissitudes of atmospheric temperature and humidity. Several times a thaw has advanced to a certain degree, with clouds from the south west, affording rain or rain and snow blended, when suddenly the wind has veered to the west or north-west, and the cold has become intense. The effect of these sudden changes, with a low average temperature, commencing, indeed, in the previous month, has been to excite in the systems of our citizens, a high degree of phlogistic diathesis, accompanied, in many of them, with actual inflammation of an acute character. At a certain period of the month, it was observed in the Cincinnati hospital, that patients affected with chronic diseases, which do not, in common, demand the lancet, required blood-letting, to give efficacy to the treatment under which they had been previously placed. All the facts indeed, which have fallen under our observation for the last six or eight weeks, indicate an absence of those mysterious atmospheric constitutions, which predispose to diseases of an asthenic or typhous character; and equally establish the positive presence of an opposite state of the atmosphere. Now, assuming that this condition of the air and the constitutions of men, is not limited to Cincinnati, but extends throughout the West, we have a favorable starting point, for observations on the course of the weather and diseases of different places for the year which is now commencing; and we take this mode and opportunity, of proposing to our readers, throughout the Valley of the Mississippi, to unite with us in simultaneous remarks on the temperature, wind, rain, snow and other characteristics of the weather, and on the prevalent diseases, of the year 1837. The dependence of the latter upon the former should constitute a special object of attention; and every thing that might throw even a ray of light, upon the causes which originate a new epidemic, or modify the character of one previously existing should be observed with perspicacity and noted down with fidelity. At the end of the year, we propose to compare all the communitions we may receive, and digest them into one report, for insertion in the Journal. In reference to the object here proposed, we beg leave to commend to our younger readers, a careful study of the works of Sydenham, being fully convinced that his writings on the appearance and disappearance of different fevers, and on what he denominates epidemic constitutions, are the very best that have been ever made, and constitute a model for general imitation.

